# Methylation and Expression of Retinoblastoma and Transforming Growth Factor-**β**1 Genes in Epstein-Barr Virus-Associated and -Negative Gastric Carcinomas

**DOI:** 10.1155/2012/906017

**Published:** 2012-09-12

**Authors:** Xia Liu, Xiuming Tang, Shaoyan Zhang, Yun Wang, Xiaofeng Wang, Chengquan Zhao, Bing Luo

**Affiliations:** ^1^Department of Medical Microbiology, Qingdao University Medical College, 38 Dengzhou Road, Qingdao 266021, China; ^2^Department of Central Laboratory, The Affiliated Hospital of Qingdao University Medical College, 19 Jiangsu Road, Qingdao 266003, China; ^3^Department of Laboratory, The Affiliated Hospital of Qingdao University Medical College, Qingdao University, 19 Jiangsu Road, Qingdao 266003, China; ^4^Department of Pathology, University of Pittsburgh Medical Center, Pittsburgh PA 15213, USA

## Abstract

*Background*. Retinoblastoma (*RB*) and transforming growth factor-**β**1 (*TGF-**β**1*) are important tumor-related factors. *Methods*. A series of 30 EBV-associated gastric carcinoma (EBVaGC) and 38 matched EBV-negative gastric carcinoma (EBVnGC) tissues were examined for the promoter methylation of *RB* by methylation-specific PCR (MSP) method. The expression of *RB* and *TGF-**β**1* in gastric carcinoma tissues was detected by immunohistochemistry. *Results*. The methylation rate of *RB* gene in EBVaGC and EBVnGC was 80.0% (24/30) and 50.0% (19/38), respectively. The difference of *RB* methylation rate between EBVaGC and EBVnGC was significant (*χ* ^2^ = 6.490, *P* = 0.011). There was no significant difference for *RB* expression between EBVaGC (43.3%, 13/30) and EBVnGC (63.2%, 24/38), and also for *TGF-**β**1* between EBVaGC (56.7%, 17/30) and EBVnGC (63.2%, 24/38). *RB* methylation was not reversely correlated with *RB* expression in gastric carcinoma tissues (*χ* ^2^ = 2.943, *P* = 0.086, *r* = 0.208). *RB* methylation, loss expression of *RB*, and *TGF-**β**1* expression were significantly associated with tumor invasion and lymph node metastasis (*P* < 0.05), but was not associated with sex, age, histological subtype (differentiation status) and tumor location. *Conclusions*. Methylation of *RB* is a common event in gastric carcinomas and EBV induces methylation of *RB* in EBVaGC, which may contribute to the development of gastric carcinomas. EBV has no significant effect on induction of *TGF-**β**1* expression. Detection of *RB* methylation, *RB* expression, and *TGF-**β**1* expression may be helpful to judge the status of tumor invasion and lymph node metastasis in gastric carcinomas.

## 1. Introduction

Gastric carcinoma is the second leading cause of cancer-related death worldwide [1]. Epstein-Barr virus (EBV) is a tumor-related herpes virus associated with the transformation of various types of cells, such as lymphoid, dendritic, smooth muscle, and epithelial cells [2]. EBV-associated gastric carcinoma (EBVaGC) is characterized by the monoclonal growth of EBV-infected epithelial cells, and the entity was recognized by Imai in 1994 [3]. EBVaGC is distributed worldwide with an annual incidence of more than 90,000 patients (10% of total gastric carcinoma (GC)) [4]. Following infection, EBV remains in a latent state in EBVaGC, which is classified as latency I. Compared with EBV-negative gastric carcinoma (EBVnGC), EBVaGC has unique clinical and pathological features, such as a younger age of incidence, high incidence in men than in women, and more diffuse than intestinal types [5–7], suggestive of a particular oncogenic mechanism of EBVaGC.

Epigenetic alterations, including methylation of CpG dinucletides in promoters and changes in chromatin structure, can affect gene expression without modifying the underlying in genetic sequences. Aberrant methylation of promoters in tumor-related genes is now regarded as one of the major mechanisms in the development of gastric carcinoma [8]. Tumor-related genes *p16*, *p14, E-cadherin, PTEN* (phosphatase and tensin homolog deleted on chromosome ten), *RASSF1A* (Ras association domain family 1A), *GSTP1* (Glutathione S-transferase pi 1), *MGMT* (O (6)-methylguanine-DNA-methyltransferase), and *MINT2 *(Munc18-1-interacting protein 2) are hypermethylated in EBVaGC [9–11], suggesting that EBV-related aberrant methylation may play an important role in development of EBVaGC.

Retinoblastoma (*RB*) and transforming growth factor-*β*1 (*TGF-*β*1*) are important regulatory factors in cell growth and differentiation, whose abnormal transcription or expression are closely associated with tumor occurrence and development. *RB* was the first successfully cloned human tumor suppressor gene (TSG). Its inactivation may result in cell proliferation leading to tumorigenesis [12]. *TGF-*β*1* is a multifunctional cytokine and triggers an intracellular signal transduction protein to regulate numerous developmental and homoeostatic processes via regulation of gene induction. It plays a dual regulatory role in cell proliferation and differentiation. In the early stage of cancer, *TGF-*β*1* can inhibit cell proliferation through arrest in the G1 phase and be regarded as a tumor suppressor; in the late stages, *TGF-*β*1* becomes a tumor promoting factor by stimulating angiogenesis, cell spread, immune suppression, and synthesis of extracellular matrix [13–16]. It has been reported that EBV latent membrane protein 1 (LMP1) has a resistant to the *TGF-*β*1-*mediated growth inhibition in EBV-positive gastric carcinoma cell lines (GT38 and GT39) and indicated that *TGF-*β*1* may be a key factor for EBV reactivation and selective growth of EBV-infected epithelial cells in vivo [17, 18]. However, the LMP1 expression is absent in EBVaGC tissues [19]. The identified role of TGF-*β*1 in EBVaGC has not been understood well and needs further research.

The absence of *RB* expression and overexpression of *TGF-*β*1* have been found in gastric carcinomas [20, 21], and the mutations and methylation of *RB* gene in gastric carcinomas were also reported in the literature [22, 23]. Mukherjee et al. [24] found *TGF-*β*1* treatment in late G(1) acutely blocks S-phase entry, this acute block by requiring the function of *RB *and loss of RB abrogates late-G(1) arrest by *TGF-*β*1*, suggesting a novel role for *RB* in mediating this effect of *TGF-*β*1* late-G(1) arrest through direct interaction with and control of the MCM helicase. However, there is no report about the expression and promoter methylation status of *RB* and *TGF-*β*1* in EBVaGC and EBVnGC to our knowledge. In this study, we examined *RB* methylation status,* RB* and *TGF-*β*1* protein expression in EBVaGC and matched EBVnGC. The aim of the study is to understand the relationship among EBV, *RB* and *TGF-*β*1* and their role in gastric carcinoma tumorgenesis. 

## 2. Materials and Methods

### 2.1. Patients and Tissue Samples

Fresh and paraffin-embedded gastric carcinoma tissues were obtained from 1678 gastric carcinoma patients in Shangdong Province, China from 2001 to 2009. The positivity of EBV in GC tissues was determined by EBV-encoded small RNA 1 in situ hybridization, as described previously [25]. The clinical features (gender, age, pathologic grade, location, invasion and lymph node metastasis) matched 30 EBVaGC and 38 EBVnGC samples were chosen for study. The study was approved by the Medical Ethics Committee at the Medical College of Qingdao University, China, and informed consent was received from all patients. 

### 2.2. DNA Extraction

DNA was extracted from fresh tumor tissues using the standard method with proteinase K digestion and phenol-chloroform purification. The QIAamp DNAFFPE Tissue kit (QIAGEN GmbH, Hilden, Germany) was used to extract the DNA from paraffin-embedded tumor tissues.

### 2.3. Immunohistochemistry (IHC)

Paraffin sections were deparaffinized and hydrated as per routine. Rabbit antihuman polyclonal antibody *TGF-*β*1* and mouse antihuman monoclonal antibody *RB* (ZSGB-Bio) were diluted to 1 : 50. The reagents (PV9000 and DAB) were obtained from ZSGB-Bio and staining was performed as per protocol. PBS (phosphate buffer saline) was used in replacement of primary antibody as a blank control. The section was considered as expressing the protein if cellular staining ≥5%, following the methods described previously [26, 27]. 

### 2.4. Bisulfite Treatment of Genomic DNA and Methylation-Specific PCR (MSP)

5 *μ*g DNA was denatured in 33.3 *μ*L of 0.3 mol/L NaOH at 37°C for 15 minutes. Denatured DNA was mixed directly with 333 *μ*L of bisulfite solution and treated in darkness. The bisulfite solution was prepared as either 2.4 mol/L sodium metabisulfite (pH 5.0–5.2) (Sigma S-1516, St. Louis, MO, USA)/0.5 mmol/L hydroquinone (Sigma H-7148) for a 4-hour treatment [28]. DNA was desalted and purified using the QIAEX Gel Extraction system (QIAGEN, Cat. no.20021). DNA was then treated with 0.3 mol/L NaOH at 37°C for 15 minutes and precipitated with 3 mol/L ammonium acetate (pH 7.0) and 1 mol/L sodium acetate (pH 5.2). Recovered DNA was dissolved in 100 *μ*L of TE buffer (pH 8.0) and stored at −20°C.

*RB *promoter methylation status was determined using MSP. In this method, bisulfite treatment converts unmethylated cytosine to uracil, but does not affect the methylated cytosine. Thus, PCR primers can be designed that anneal selectively to methylated or unmethylated DNA after bisulfite conversion. The sequences of the unmethylated DNA and methylated DNA-specific primers are listed in Table 1. The primer UF/UR pair was designed specifically for amplification of the bisulfite-converted unmethylated promoter, while the MF/MR primer pair was designed specifically for the amplification of the bisulfite-converted methylated promoter. MSP results determined whether the samples are methylated or unmethylated. If there is M primers amplified band, the sample was considered to be in the methylation status. One microliter of bisulfite-treated DNA (around 25 ng) was amplified with 1.5 mmol/L MgCl_2_ and 0.2 mmol/L dNTP in a 25 *μ*L reaction volume. Primers were used at a final concentration of 0.4 mmol/L each. The PCR involved an initial denaturation at 94°C for 10 minutes, followed by 40 cycles consisting of 94°C for 30 seconds, predetermined optimal annealing temperature for 30 seconds, 72°C for 30 seconds, and a final extension at 72°C for 5 minutes. Eight microliters of PCR product were analyzed on a 2.0% agarose gel. Water was used as a negative control.

### 2.5. Statistical Analysis

*RB* promoter methylation status, *RB* expression and *TGF-*β*1* expression between EBVaGC and EBVnGC was compared using the Chi-square test. The correlation between promoter methylation and the protein expression was analyzed by Paired fourfold table Chi-square test. The association of clinical features with *RB* promoter methylation, *RB* expression, and *TGF-*β*1* expression was compared by chi-square test. *P* < 0.05 was considered to be statistically significant.

## 3. Results

### 3.1. Comparison of Clinicopathological Data between EBVaGC and EBVnGC Patients

102 of 1678 (6.1%) cases of gastric carcinoma were EBV positive. 30 EBVaGC and 38 EBVnGC tumor tissues with matching clinical parameters were chosen for methylation detection. The clinical and pathological data are listed in Table 2. The two kinds of gastric carcinomas were similar in gender, age, pathologic grade, location, invasion, and lymph node metastasis.

### 3.2. The Promoter Methylation Status of *RB* Gene in EBVaGC and EBVnGC

**Figure 1:**
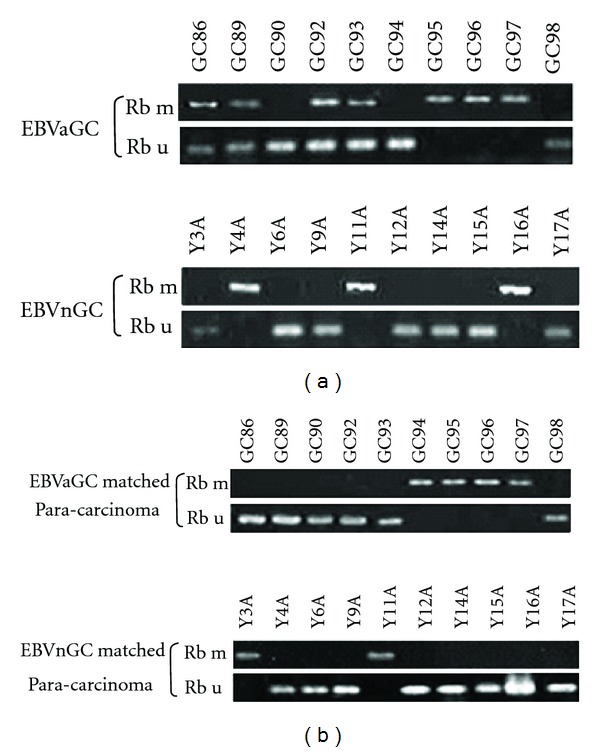
*RB* promoter methylation in EBVaGC, EBVnGC, and matched paracarcinoma tissues. (a) Representative *RB* promoter methylation in EBVaGC and EBVnGC by MSP. U, PCR product from the MSP assay using primers specific for the unmethylated allele; M, PCR product from the MSP assay using primers specific for the methylated allele. (b) Representative *RB* MSP assay results for matched paracarcinoma tissues.

Promoter methylation of the *RB *gene was detected by MSP (Figure 1(a)). In total, 43/68 (63.2%) cases of gastric carcinomas demonstrated *RB* gene promoter methylation. The difference in the percent of positive methylation bands detected by MSP for *RB* was statistically different between EBVaGC (24/30, 80%) and EBVnGC (19/38, 50.0%) (*P* = 0.011). 

The association of clinicopathological parameters of 68 cases with *RB* gene methylation status was studied. There was no relationship of *RB* gene methylation status with patient age, gender, pathologic types, and tumor location. However, the methylation status was associated with the depth of tumor invasion (*P* = 0.036) and lymph node metastasis (*P* = 0.012), (Table 3). 

### 3.3. The Promoter Methylation Status of *RB* Gene in GC and Corresponding Adjacent Normal Gastric Tissues

The promoter methylation of *RB* gene was detected in EBVaGC and EBVnGC corresponding adjacent normal gastric tissues (Figure 1(b)). The percent of positive methylation bands by MSP for *RB* in gastric carcinoma and corresponding adjacent normal gastric tissues was 63.2% (43/68) and 39.7% (27/68); the difference was significant (*P* = 0.006).

### 3.4. The Protein Expression of *RB* and *TGF-*β**1

**Figure 2:**
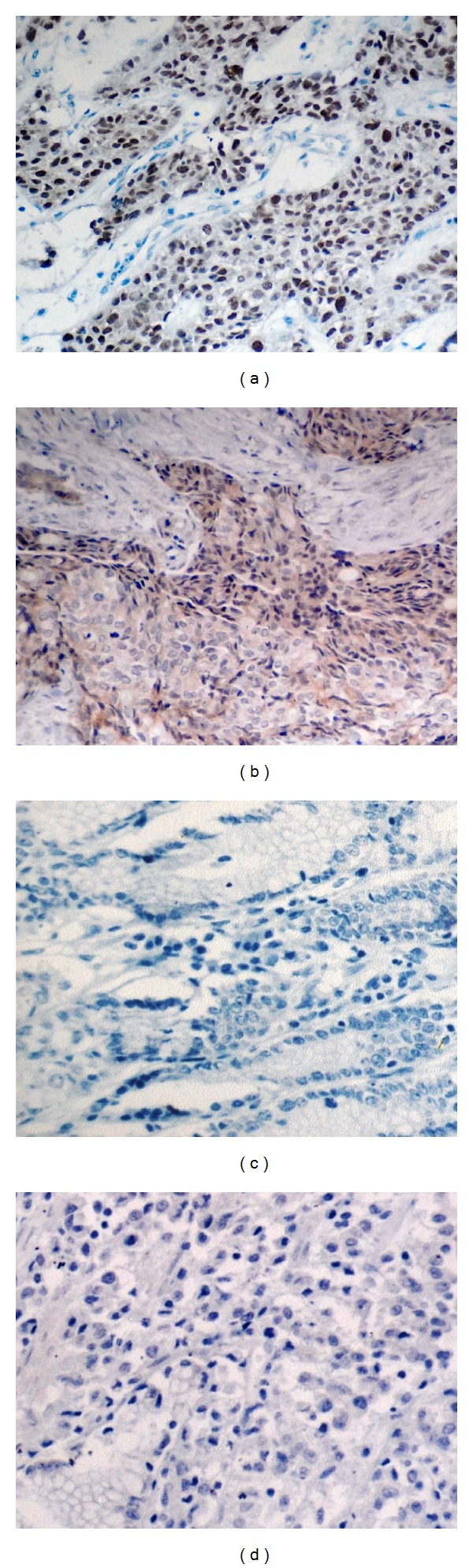
The protein expression of *RB *and *TGF-*β*1* by immunohistochemistry (magnification ×100). (a) Positive immunohistochemistry result of *RB* in paraffin section. Expression of *RB* was found in nuclei of gastric carcinoma cells. (b) Positive immunohistochemistry result of *TGF-*β*1* in paraffin section. Expression of *TGF-*β*1* was found in cytoplasm of gastric carcinoma cells. (c) Negative immunohistochemistry result of *RB* in paraffin section. (d) Negative immunohistochemistry result of *TGF-*β*1* in paraffin section.

*RB* and *TGF-*β*1* protein expression was detected by IHC, shown in Figures 2(a) and 2(b). The percent of EBVaGC that were positive by IHC for *RB* was 43.3% (13/30), lower than in EBVnGC (63.2%, 24/38), but not significantly different (*P* = 0.103). There was not obvious difference of *TGF-*β*1 *protein expression between EBVaGC (56.7%, 17/30) and EBVnGC (63.2%, 24/38) (*P* = 0.587) (Table 4).

The correlation of *RB *protein expression with *RB *promoter methylation was studied. There were 23 negative *RB* protein expression cases in 43 *RB* gene methylated gastric carcinoma (53.5%), which was higher than in *RB* gene unmethylated gastric carcinoma (32.0%, 8/25), but without reverse correlation (*P* = 0.09, *r* = 0.21), (Table 5). 

The association between *RB* expression and clinicopathological parameters is shown in Table 6. There was no relationship between *RB* protein expression and patients' age, gender, pathological grade, and tumor location, but it was related with the depth of tumor invasion (*P* = 0.04) and lymph node metastasis (*P* = 0.02).

The association between *TGF-*β*1* expression and clinicopathological parameters is shown in Table 6. There was no relationship between *TGF-*β*1* protein expression and patients' age, gender, pathological types, and tumor location, but there was positive association between *TGF-*β*1* protein expression and the depth of tumor invasion (*P* = 0.02) and lymph node metastasis (*P* = 0.002).

## 4. Discussion

In this study, the percent of EBVaGC with positive methylation bands by MSP for *RB* was significantly higher than that of EBVnGC, indicating that EBV may induce *RB* promoter methylation during infection. Previous studies showed that EBVaGC had higher methylation frequency and promoter CpGI methylation density than EBVnGC in some TSGs, such as p16, E-cadherin and p73, and the methylation status was reverse correlated with protein expression [4, 10, 29]. These results indicate that methylation and silence of TSGs induced by EBV may be an important oncogenic mechanism for the development of EBVaGC. Only a few studies detected the methylation of *RB* in gastric carcinomas. Zhao et al. [30] found that the percent of positive methylation bands for *RB* gene was 44.6% (45/101), similar to our study of 38 EBVnGC (50%), but less than that of 30 EBVaGC cases (80%), which provides further support that EBV induces *RB* gene methylation in EBVaGC. 

Promoter CpG island methylation is considered an important mechanism of TSG inactivation. In the present study, 23 of 43 (53.5%) methylated gastric carcinoma tissues lost* RB* protein expression, which was higher than that in unmethylated gastric carcinoma tissues (32.0%, 8/25), but *RB *promoter methylation was not reversely correlated with* RB *protein expression (*P* = 0.086). This phenomenon was also found between *p16INK4* gene methylation status and expression in meningiomas by Tse et al. [31]. The possible explanations include: (1) gene methylation in gastric carcinoma tissue is heterogeneous; (2) gene methylation may occur in only one allele of cancer cells, while the other allele remains unmethylated. The above reasons may also explain the existence of methylated and unmethylated gene bands by MSP. Increasing of CpG island methylation density is a dynamic process, and only the methylation density increases to a certain extent, it results in the complete loss of the expression. The *RB* promotor methylation could result the decrease or loss of protein expression. Thus, lacking reverse correlation between *RB* promotor methylation and protein expression was not contradictory. Because of the limitation of major disadvantage of MSP, the methylation status of single CpG site in primer binding sequences is not be detected [32]. The correlation between *RB* promoter methylation dynamic change and protein expression need further study. These reasons above can also be used to explain why there wasn't a significant difference in *RB* protein expression between EBVaGC and EBVnGC, even though *RB* promoter methylation of EBVaGC was significantly higher than EBVnGC. If the *RB* gene promoter methylation and its protein expression were negatively correlated, *RB* protein expression in EBVaGC should have been significantly lower than that in EBVnGC. In this study, the percent of EBVaGC and EBVnGC that were positive by IHC for *RB* were 43.3% and 63.2%, respectively. Although no significant difference was found of the positive rate of *RB* protein expression between EBVaGC and EBVnGC, the relatively lower expression rate in EBVaGC also suggests that EBV-induced *RB* promoter methylation could lead to inhibition of *RB* protein expression to some extent. Moreover, the promoter methylation of *RB* gene was also detected in GC and corresponding adjacent normal gastric tissues and the difference was significant, which confirmed that RB promoter methylation is involved with the development of GC. 

Similar to the result of *RB* protein expression, the positive rate of* TGF-*β*1* protein expression was 56.7% (17/30) and 63.2% (24/38) in EBVaGC and EBVnGC, respectively, without significant difference (*P* = 0.404), suggesting that EBV is not related to the *TGF-*β*1 *expression in EBVaGC. Kim et al. [33] examined the association of EBV with *RB* and *p53 *protein expression in classic Hodgkin lymphoma and found that EBV wasn't associated with *RB* and *p53 *protein expression. Xu et al. [34] found *TGF-*β*1* level in the serum of nasopharyngeal cancer patients was significantly higher than that in normal persons, and also the advanced stage was higher than the early stage, and recurrent tumors was higher than primary tumors, which indicating that serum *TGF-*β*1* can be used for diagnosis and judgment for prognosis of NPC, and EBV infection can induce the synthesis and release of *TGF-*β*1*. This result was different from our result of *TGF-*β*1 *in EBVaGC.

Previous studies showed that *TGF-*β*1* expression rate and expression level were higher in gastric carcinoma tissues than that in normal tissues, and the *TGF-*β*1* expression were associated with gastric invasion, metastases, and prognosis [35–37]. In the advanced cancer, *TGF-*β*1 *can provide the microenvironment suitable for tumor growth, invasion, and metastases by stimulating angiogenesis, cell spread, immune suppression, and synthesis of extracellular matrix. In gastric carcinoma, *RB* protein loss was also found to be associated with metastases and prognosis [38–41]. The *RB* protein expression rate was 40% ~ 90% [38–40, 42, 43] and *TGF-*β*1* protein expression rate was 22.8% ~ 71% [35–37, 44] in previous studies. In the present study, *RB* protein loss and *TGF-*β*1* protein wasn't associated with patient age, gender, pathologic types, and tumor location, but associated with the depth of tumor invasion and lymph node metastasis. At the same time, we also confirmed that *RB* promoter methylation was associated with tumor invasion and lymph node metastasis, indicating that *RB* promoter methylation, *RB *and* TGF-*β*1 *protein expression can be as clinical reference index for judgement of gastric carcinoma invasion and metastasis. 

## 5. Conclusion 

Our study showed that Aberrant *RB* promoter methylation was common in gastric carcinoma. EBV could induce *RB* gene methylation and affect the gene expression in EBVaGC development. EBV has no significant effect on *TGF-*β*1 *expression. 

## Figures and Tables

**Table 1:** List of primers used in MSP.

Primers	Sequence	Product size (bp)	Annealing temp (0°C)	Genomic position
*RB*MF	5′GGGAGTTTCGCGGACGTGAC3′	163	60	−61 to 102
*RB*MR	5′ACGTCGAAACACGCCCCG3′			
*RB*UF	5′GGGAGTTTTGTGGATGTGAT3′	163	58	−61 to 102
*RB*UR	5′ACATCAAAACACACCCCA3′			

**Table 2:** Comparison of clinicopathological data between EBVaGC and EBVnGC patients.

	EBVaGC (*n* = 30)	EBVnGC (*n* = 38)	*χ* ^ 2^	*P*
Age (yr)				
<50	18	19	0.676	0.411
≥50	12	19		
Gender				
Male	27	31	—	0.494
Female	3	7		
Pathologic grade				
Poorly differentiated	28	32	—	0.288
Well-moderately differentiated	2	6		
Location				
Gastric cardia	7	7	—	0.738
Gastric body	13	15		
Antrum	10	16		
Depth of invasion				
Invasion to serosa and invasion through serosa	22	24	0.793	0.373
Not invading serosa	8	14		
Lymph node metastasis				
Positive	17	21	0.134	0.908
Negative	13	17		

**Table 3:** Correlation of methylation status of *RB* gene with clinicopathological data of gastric carcinoma patients.

	*n*	Methylated (*n*)	Unmethylated (*n*)	*χ* ^ 2^	*P*
EBV infection					
EBVaGC	30	24	6	6.490	0.011
EBVnGC	38	19	19		
Age (yr)					
<50	37	22	15	0.498	0.481
≥50	31	21	10		
Gender					
Male	58	36	22	—	0.835
Female	10	7	3		
Pathologic grade					
Poorly differentiated	60	40	20	—	0.216
Well-moderately differentiated	8	3	5		
Location					
Gastric cardia	14	10	4	3.172	0.205
Gastric body	28	20	8		
Antrum	26	13	13		
Depth of invasion					
Invasion to serosa and invasion through serosa	46	33	13	4.423	0.036
Not invading serosa	22	10	12		
Lymph node metastasis					
Positive	38	29	9	6.339	0.012
Negative	30	14	16		

**Table 4:** Comparisons of the expression of *RB* and *TGF-*β*1* between EBVaGC and EBVnGC.

	*n*	*RB* expression		*TGF-*β*1 *expression	
		Positive	Negative	Positive	Negative
EBVaGC	30	13	17	17	13
EBVnGC	38	24	14	24	14
*χ* ^ 2^		2.656		0.295	
*P*		0.103		0.587	

**Table 5:** Correlation of methylation status of *RB* gene with its protein expression.

Methylation status	Protein expression		Total
	Positive	Negative
Methylated	20	23	43
Unmethylated	17	8	25

Total	37	31	68

**Table 6:** Correlation of expression of *RB* an*d TGF-*β*1* protein with clinicopathological data of gastric carcinoma patients.

	*n*	*RB*				*TGF-*β*1*			
		Expression (*n*)	Absent (*n*)	*χ* ^ 2^	*P*	Expression (*n*)	Absent (*n*)	*χ* ^ 2^	*P*
Age (yr)									
<50	37	21	16	0.180	0.671	20	17	1.320	0.251
≥50	31	16	15			21	10		
Gender									
Male	58	31	27	—	0.972	35	23	—	0.742
Female	10	6	4			6	4		
Pathologic grade									
Poorly differentiated	60	31	29	—	0.428	38	22	—	0.295
Well-moderately differentiated	8	6	2			3	5		
Location									
Gastric cardia	14	7	7	2.091	0.352	8	6	0.318	0.853
Gastric body	28	13	15			18	10		
Antrum	26	17	9			15	11		
Depth of invasion									
Invasion to serosa and invasion through serosa	46	21	25	4.398	0.036	32	14	5.105	0.024
Not invading serosa	22	16	6			9	13		
Lymph node metastasis									
Positive	38	16	22	5.259	0.022	29	9	9.235	0.002
Negative	30	21	9			12	18		

## Abbreviations

GC: Gastric carcinoma
EBV: Epstein-Barr virus
EBVaGC: Epstein-Barr virus-associated gastric carcinoma
EBVnGC: EBV-negative gastric carcinoma
TSG: Tumor suppressor gene
*RB*: Retinoblastoma
*TG**F*-*β*1: Transforming growth factor-*β*1.
